# Assessing observer-dependent dental age estimation procedures: intra- and inter-observer reliability across four well established radiographic systems for dental analysis

**DOI:** 10.1007/s00414-025-03616-w

**Published:** 2025-10-25

**Authors:** Nikolaos Angelakopoulos, Rizky Merdietio Boedi, Ademir Franco, Nikita Polukhin, Akiko Kumagai, Ivan Galic, Jeta Kelmendi, Israel Soriano Vázquez, Sang-Seob Lee, Galina Zolotenkova, Roberto Scendoni, Stefano De Luca

**Affiliations:** 1 Department of Orthodontics and Dentofacial Orthopedics, University of Bern, Bern, Switzerland; 2 Department of Forensic Dentistry, Faculdade São Leopoldo Mandic, Campinas, Brazil; 3 Department of Dentistry, Faculty of Medicine, Universitas Diponegoro, Semarang, Indonesia; 4 Department of Public Health and Medical Social Sciences, Synergy University, Moscow, Russian Federation; 5 Department of Forensic Science, Division of Forensic Odontology and Disaster Oral Medicine, Iwate Medical University, Iwate, Japan; 6 Department of Oral Surgery, School of Medicine, University Hospital of Split, University of Split, Split, Croatia; 7 Department of Orthodontics, University Dentistry Clinical Center of Kosovo, Pristina, Kosovo; 8 Forensic Odontologist, independent researcher, Mexico City, Mexico; 9 Department of Anatomy, Catholic Institute for Applied Anatomy, College of Medicine, The Catholic University of Korea, Seoul, Seocho-gu Republic of Korea; 10 Department of Forensic Medicine, I.M. Sechenov First Moscow State Medical University, Moscow, Russian Federation; 11 Department of Law, Institute of Legal Medicine (AgEstimation Project), University of Macerata, Macerata, Italy; 12 Department of Organisms and Systems Biology, Faculty of Biology, University of Oviedo, Oviedo, Spain

**Keywords:** Forensic odontology, Dental age estimation, Reliability, Third molar, Panoramic radiographs

## Abstract

In forensic contexts, age assessments constitute matters of substantial legal consequence, particularly in proceedings involving children and young adolescents. Dental age estimation (DAE) techniques are widely used for this purpose, especially in cases involving undocumented minors. This study assesses intra- and inter-observer reliability across four well established radiographic systems for dental analysis.: Gleiser and Hunt Modified by Köhler (GHK), Demirjian (DEM), Kullman (KUL), and Cameriere’s Third Molar Maturity Index (I3M). A total of 50 panoramic radiographs from individuals aged 14-23.99 years were analyzed by nine qualified forensic experts. The observers assessed the development stages of third molars using the three staging methods (GHK, DEM, KUL) and measured the I3M using Cameriere's metric approach. Primarily, the quantitative assessment for analyzing the agreement was Cohen’s Kappa, Gwet’s Agreement Coefficient (AC1) and (AC2), and Intraclass Correlation Coefficient (ICC). Statistical analysis revealed high intra-observer reliability for all methods, with coefficient values indicating strong agreement among individual observers. In terms of inter-observer reliability, the I3M achieved the highest agreement (ICC 0.986), followed by DEM (AC2 0.918), GHK (AC2 0.914), and KUL (AC2 0.868). Notably, maxillary third molars consistently showed lower inter-observer agreement than mandibular third molars, particularly when assessed using the DEM and GHK methods. The highest inter-observer agreement in cases where a tooth could not be staged or measured was observed for the KUL method (AC1 0.993), followed by I3M (AC1 0.988), with DEM and GHK, demonstrating equivalent levels of agreement (AC1 0.954). All of the tested methods yielded highly reliable results, especially DEM and GHK. The choice of a staging method should be guided by the specific objectives of each study. Moreover, while the I3M method demonstrated high reliability values, obtaining identical repeated measurements was nearly impossible due to its metric approach.

## Introduction

Age estimation in living individuals carries significant legal and humanitarian implications. This process of estimating an individual’s age plays a crucial role in various contexts, including, but not limited to, criminal proceedings, immigration cases, competitive sports, and human trafficking [1–6]. By estimating an individual’s age, especially when they do not possess proper legal identity documents (i.e., passports, birth certificates), the individual may gain access to benefits or justice based on their age. Although multiple age estimation methods can be applied across various age ranges, children and juveniles have become a primary focus of forensic age estimation due to the frequent association with undocumented or asylum-seeking minors.

Among the various approaches available for age estimation, dental techniques are among the most widely utilized, particularly for children and juveniles [7]. In the dental age estimation (DAE) process for these age groups, radiographic analysis is typically performed to assess tooth development, with the results of the observation then being converted into an estimated dental age. Forensic institutions and expert consultants that routinely handle such cases are advised to follow the recommendations of expert groups, such as the Study Group on Forensic Age Diagnostics of the German Society of Forensic Medicine (AGFAD) [6, 8]. Others, such as the International Organization for Forensic Odonto-Stomatology (IOFOS) and the American Board of Forensic Odontology (ABFO), also emphasize the need for methodological rigor. Specifically, these groups recommend that (1) more than one statistically independent DAE method be applied, and (2) two independent observers conduct the assessment, with any discrepancies resolved accordingly [9, 10].

Given these considerations, multiple DAE methods can be employed to estimate an individual’s age, each method exhibiting varying degrees of reproducibility [11]. The reproducibility and reliability of these age estimation methods, whether based on the graded interpretation of dental radiographs or a quantitative analysis of third molar mineralization, hinge on the repeatability of results and the agreement between observers. This agreement is crucial when a radiograph is analyzed by multiple observers (inter-observer reliability) or when it is re-examined by the same observer at different times (intra-observer reliability) [12].

While the accuracy of a DAE method can be determined through robust statistical evaluation (i.e., cross-validation [13], and balanced sample dispersion across age ranges and sexes) [14], the reliability of the method itself in producing the model is equally critical. This issue often arises from the inherently subjective nature of the DAE process, which may lead to discrepancies in the results [15]. This study aimed to compare the reliability of four well established radiographic systems for dental analysis in order to assess their consistency. By evaluating intra- and inter-observer reliability, the study seeks to determine the level of agreement across these methods and identify potential sources of variation.

## Materials and methods

### Study sample

This observational, cross-sectional study was designed to assess intra- and inter-observer reliability in age estimation across four well-established DAE methods using panoramic radiographs.

A sample of 50 anonymized panoramic radiographs from 50 subjects aged between 14 and 23.99 years was selected, including 19 males and 31 females. These panoramic radiographs were selected from a previously established, anonymized dataset that has been utilized in prior scientific publications. No new data collection, database access, patient contact, or subjects exposed to radiation solely for the purpose of this research. Anonymization of the panoramic radiographs was achieved by extracting only the data relevant to this study, including the radiograph identification number, sex, date of birth, and date of exposure.

The number of samples was established using a sample size determination based on precision rather than to test a null hypothesis rejection due to no standardized procedure for Gwet’s AC power analysis. Calculations were performed in accordance with Rotondi and Donner (2012) [16] using the kappaSize R package, originally developed for Cohen’s and Fleiss’ kappa sample size estimation. It was assumed as an appropriate approach since it was reported that Gwet’s AC yields lower variance, providing greater precision and potentially requiring smaller sample sizes than other reliability coefficients [17].

The kappaSize package limits settings to 5 categories and 6 observers, so the performed estimation can be considered as conservative, since increasing the number of categories and observers reduces the required sample size [16]. The expected reliability coefficient value was set conservatively at 0.8, based on the previously conducted study results obtained in similar settings [18] with desirable half-width of the confidence interval of 0.1, less strict than in clinical contexts to achieve a balance between feasibility and precision. Estimates under both uniform and skewed distributions were considered, and the larger result was adopted, yielding a sample size of 48.

For the intraclass correlation coefficient (ICC), a similar rationale was applied along with parameters: expected ICC value of 0.8, desirable half-width of the confidence interval of 0.1 but with an exact number of observers (9). The method described by Bonnet [19] was adopted, resulting in a requirement of a minimum of 25 observations.

Inclusion criteria included high-quality panoramic radiographs showing all four third molars in intact condition. Exclusion criteria involved panoramic radiographs displaying dental abnormalities, supernumerary teeth, or any conditions that could interfere with normal dental development, tumors, surgical materials, mandibular or maxillary fractures, gross pathology, history of orthodontic treatment, or signs of infection in the third molar regions. Additionally, radiographs of poor quality that hindered accurate interpretation, unclear radiographs with radiographic distortion, individuals with a history of third molar extraction, those with primarily retained third molars, and cases of third molar agenesis were excluded from the analysis. None of the X-rays were taken specifically for this research.

### Data management

All third molars were numbered according to the two-digit FDI (*Fédération Dentaire Internationale*) system: 18 for the right maxillary third molar, 28 for the left maxillary third molar, 38 for the left mandibular third molar, and 48 for the right mandibular third molar [20]. Each panoramic radiograph was anonymized and assigned a unique identification number. To determine the chronological age (CA) of each subject, the date of birth was subtracted from the date of radiograph exposure. Detailed information for each radiograph, including identification number, sex, date of birth, date of radiograph exposure, and CA, was meticulously recorded using Microsoft Excel 2016 for data management. The study was carried out under the ethical standards laid down by the Declaration of Helsinki (Finland) and its later amendments [21].

### Assessment systems

For this study, four DAE methods were selected: three staging techniques—Gleiser and Hunt as modified by Köhler (GHK) [22, 23], Demirjian et al. (DEM) [24], and Kullman (KUL) [25]—and one metric approach, Cameriere et al.’s Third Molar Maturity Index (I3M) [26]. These methods were selected based on their widespread use, their simplicity, robustness, validation across multiple populations, or a combination of these factors [27].

### Participants and calibration

A total of nine observers participated in the study, comprising five qualified forensic odontologists with expertise in DAE and four forensic experts with previous experience in forensic DAE and publications (Table 1). DAE research experience was indicated through academic publications with the DAE as a main topic. Clinical DAE cases experience was indicated through each of the observer’s experiences in dealing with real-world DAE cases (i.e., identification, Disaster Victim Identification, asylum seeker age assessments). Each observer independently applied all four methods to the selection of 50 radiographs.

**Table 1:** Summary of observers’ experience based on years of expertise, research case studies, and clinical dental age estimation evaluations

Observer	Experience (Years)	DAE Research Experience	Clinical DAE Cases Experience
1	9	Yes	Yes
2	6	Yes	Yes
3	25	Yes	Yes
4	14	Yes	Yes
5	10	Yes	Yes
6	13	Yes	Yes
7	28	Yes	Yes
8	10	Yes	Yes
9	13	Yes	Yes

Calibration instructions were provided to each observer to ensure a standardized application of each method. The guidelines were as follows:

1. In cases of uncertainty regarding which stage to choose, the observer was instructed to select the lower stage.
2. For multi-rooted teeth exhibiting differing stages of development (e.g., the distal root at stage G and the mesial root at stage H), the lower stage was to be taken into consideration.
3. No changes were to be made to the opposite side if the tooth could not be observed.
4. In situations where a tooth could not be staged, measured, or visualized correctly due to image distortion, observers were instructed to mark “Not Applicable (NA)” in the form.
5. Observers were allowed to use any image processing software to aid in the process, with a request to inform the project supervisors of the software chosen.

Observers were blinded from any radiograph’s metadata, including CA and sex to ensure minimal bias. Inter-observer reliability was assessed based on the initial evaluations, while intra-observer reliability was determined using repeated observations. Specifically, all panoramic radiographic images were evaluated two weeks after the initial staging and measurements by each observer to determine the intra-observer agreement.

### Statistical analysis

To ensure that the selected radiograph samples achieved 80% statistical power, a simulation-based analysis was conducted using the irrCAC R package. Ordinal ratings were generated for nine observers and 7, 8, and 10 categories corresponding to the number of stages adopted in each of the tested DAE staging methods under three marginal distributions: uniform, skewed to lower stages, and skewed to larger stages. The target AC2 (expected agreement) and the null AC2 (chance agreement) were conservatively set at 0.6 and 0.4, respectively. For each condition, the correlation parameter was calibrated to achieve the target mean AC2, thereby ensuring the consistency of simulated ratings around the marginal category probabilities. Subsequently, rating matrices were simulated, AC2 coefficients were estimated, and one-sided tests were conducted. Power was defined as the proportion of simulations rejecting the null hypothesis, and the minimum sample size required to attain 80% power was extracted for each distribution and category set. A total of 25 observations was sufficient across all distributions and category sets, confirming that the planned sample size of 50 radiographs was robust.

Intra-observer and inter-observer reliability tests were selected for analysis based on the nature of the collected variables. For intra-observer reliability, weighted Cohen’s Kappa was used for ordinal variables (KUL, DEM, and GHK), while the Intraclass Correlation Coefficient (ICC) with a two-way mixed model, focusing on absolute agreement, was employed for continuous variables (I3M). Table 2 presents the absolute numbers and proportions for each category for methods employing ordinal stages, as well as the median and quartiles for the I3M method, which employs continuous data. The selection of Gwet’s AC2 (weighted test) as the preferred method for assessing inter-observer reliability of ordinal variables was based on its proven ability to overcome limitations such as the “Kappa paradox,” where weighted Fleiss' Kappa and Krippendorff’s alpha may yield low agreement values despite high observed concordance due to imbalanced category prevalence or marginal distributions. The use of the Gwet’s AC2 with the ordinal weighting technique was specifically chosen in accordance with Gwet’s recommendations (2014) [28] for ordinal categories. This approach assigns smaller penalties to minor staging differences and larger penalties to substantial discrepancies, in contrast to the unweighted Gwet’s AC1, which treats all category differences equally, as it is designed for nominal data.

**Table 2:** Categorical distributions, including Not applicable teeth (“NA”), and median values of continuous variables

Kullman, *n* (%)*			Demirjian, *n* (%)*					Gleiser and Hunt as modified by Köhler *n* (%)*					Cameriere I3M, Me (Q_1_-Q_3_)**	
	**KUL 38**	**KUL 48**		**DEM 18**	**DEM 28**	**DEM 38**	**DEM 48**		**GHK 18**	**GHK 28**	**GHK 38**	**GHK 48**	**I3M 38**	**I3M 48**
1 2 3 4 5 6 7 NA	77 (17.1%) 75 (16.7%) 58 (12.9%) 112 (24.9%) 78 (17.3%) 35 (7.8%) 14 (3.1%) 1 (0.2%)	74 (16.4%) 66 (14.7%) 66 (14.7%) 102 (22.7%) 81 (18.0%) 34 (7.6%) 24 (5.3%) 3 (0.7%)	**A** **B** **C** **D** **E** **F** **G** **H** **NA**	0 (0.0%) 0 (0.0%) 0 (0.0%) 46 (10.2%) 122 (27.1%) 135 (30.0%) 85 (18.9%) 41 (9.1%) 21 (4.7%)	0 (0.0%) 0 (0.0%) 0 (0.0%) 48 (10.7%) 124 (27.6%) 136 (30.2%) 82 (18.2%) 39 (8.7%) 21 (4.7%)	0 (0.0%) 0 (0.0%) 4 (0.9%) 74 (16.4%) 115 (25.6%) 166 (36.9%) 70 (15.6%) 21 (4.7%) 0 (0.0%)	0 (0.0%) 0 (0.0%) 3 (0.7%) 68 (15.1%) 116 (25.8%) 147 (32.7%) 82 (18.2%) 30 (6.7%) 4 (0.9%)	**1** **2** **3** **4** **5** **6** **7** **8** **9** **10** **NA**	0 (0.0%) 0 (0.0%) 5 (1.1%) 54 (12.0%) 88 (19.6%) 97 (21.6%) 69 (15.3%) 46 (10.2%) 35 (7.8%) 34 (7.6%) 22 (4.9%)	0 (0.0%) 0 (0.0%) 5 (1.1%) 57 (12.7%) 85 (18.9%) 100 (22.2%) 67 (14.9%) 49 (10.9%) 31 (6.9%) 36 (8.0%) 20 (4.4%)	0 (0.0%) 1 (0.2%) 12 (2.7%) 64 (14.2%) 101 (22.4%) 82 (18.2%) 91 (20.2%) 59 (13.1%) 23 (5.1%) 16 (3.6%) 1 (0.2%)	0 (0.0%) 1 (0.2%) 8 (1.8%) 64 (14.2%) 99 (22.0%) 86 (19.1%) 76 (16.9%) 61 (13.6%) 24 (5.3%) 27 (6.0%) 4 (0.9%)	0.27 (0.18–0.45) **NA** 1 (0.2%)	0.26 (0.17–0.42) **NA** 7 (1.6%)

*Percentages may not sum to exactly 100% due to rounding. ** Median (Quartile_*1*_*-Quartile*_*3*_*)*

To maintain a conservative weighting scheme, categories that were not presented in the evaluations of any of the observers were excluded from the weight matrix. This approach amplifies the relative penalty for discrepancies in comparison to an alternative weighing strategy that incorporates such categories. To ensure a valid and unbiased estimation of the agreement coefficients, NA cases were excluded. The total number of observations included in the evaluation were provided in each table.

Inter-observer reliability for continuous data was assessed using the ICC. Additionally, the agreement on NA responses was evaluated separately using Gwet’s AC1 (unweighted test). For this analysis, all variables were transformed into binary categories (“NA”/”Not NA”) before calculating Gwet’s AC1. The absolute number and the proportion of NA responses for each DAE method is presented in Table 2. Category prevalence analysis showed that NA responses did not exceed 5% among all observers for each tooth stage evaluation, indicating an imbalance in category distribution. Such an imbalance can result to the “Kappa Paradox”, in which agreement is underestimated despite high observed concordance. To address this, we used Gwet’s AC1, which adjusts for prevalence effects and provides more stable agreement estimates.

The benchmarking of weighted Cohen’s Kappa and Gwet’s AC1/AC2 was adapted from Landis and Koch (1977) [29] as follows: <0 = poor, 0.0-0.20 = slight, 0.21–0.40 = fair, 0.41–0.60 = moderate, 0.61–0.80 = substantial, 0.81-1.0 = almost perfect agreement. ICC was interpreted in accordance with Portney and Watkins (2000) [30]: <0.5 = poor; 0.5–0.75 = moderate, 0.75–0.9 = good, > 0.90 = excellent reliability. Confidence intervals (95%) and p-values were provided along with reliability tests coefficients.

The statistical tests were conducted using IBM SPSS 26.0 and R ver. 4.5.1 with the “irrCAC” and “kappaSize” packages.

## Results

A total of nine observers participated in the study, all of whom demonstrated consistent internal reliability. The intra-observer reliability values for staging agreement, as measured by Cohen’s Kappa, were all above 0.8, and the ICC values were close to or exceeded 0.9. However, it was noted that the maxillary third molars consistently received lower scores in both the DEM and the other staging methods (Table 3).

**Table 3:** Intra-observer reliability

Intra-observer Reliability	Agreement Value [95% CI]*											
	Kullman		Demirjian				Gleiser and Hunt as modified by Köhler				Cameriere I3M	
	KUL 38	KUL 48	DEM 18	DEM 28	DEM 38	DEM 48	GHK 18	GHK 28	GHK 38	GHK 48	I3M 38	I3M 48
Observer 1	0.965 [0.943; 0.988]	0.974 [0.954; 0.994]	0.936 [0.886; 0.986]	0.964 [0.927; 1.001]	0.939 [0.893; 0.985]	0.950 [0.913; 0.987]	0.891 [0.812; 0.970]	0.938 [0.892; 0.985]	0.945 [0.919; 0.971]	0.964 [0.943; 0.985]	0.981 [0.966; 0.989]	0.984 [0.971; 0.991]
Observer 2	0.954 [0.930; 0.979]	0.953 [0.919; 0.987]	0.884 [0.812; 0.956]	0.855 [0.771; 0.939]	0.931 [0.876; 0.986]	0.901 [0.842; 0.960]	0.908 [0.851; 0.965]	0.895 [0.831; 0.959]	0.941 [0.904; 0.977]	0.940 [0.905; 0.976]	0.993 [0.986; 0.996]	0.971 [0.948; 0.983]
Observer 3	0.926 [0.886; 0.966]	0.944 [0.909; 0.979]	0.932 [0.888; 0.976]	0.941 [0.900; 0.982]	0.930 [0.885; 0.975]	0.934 [0.889; 0.979]	0.967 [0.944; 0.99]	0.961 [0.935; 0.986]	0.958 [0.932; 0.984]	0.929 [0.885; 0.974]	0.893 [0.409; 0.963]	0.914 [0.482; 0.971]
Observer 4	0.962 [0.939; 0.985]	0.905 [0.81; 1.000]	0.881 [0.818; 0.943]	0.941 [0.893; 0.989]	0.914 [0.868; 0.961]	0.942 [0.903; 0.980]	0.884 [0.783; 0.985]	0.898 [0.808; 0.988]	0.944 [0.904; 0.985]	0.939 [0.897; 0.981]	0.993 [0.987; 0.996]	0.955 [0.918; 0.975]
Observer 5	0.989 [0.976; 1.000]	1.000 [1.000; 1.000]	1.000 [1.000; 1.000]	0.991 [0.974; 1.000]	1.000 [1.000; 1.000]	0.984 [0.962; 1.000]	0.997 [0.990; 1.000]	0.993 [0.983; 1.000]	0.996 [0.988; 1.000]	0.993 [0.984; 1.000]	0.985 [0.965; 0.993]	0.984 [0.973; 0.991]
Observer 6	0.851 [0.747; 0.955]	0.880 [0.786; 0.973]	0.904 [0.841; 0.966]	0.918 [0.864; 0.972]	0.951 [0.915; 0.988]	0.965 [0.931; 1.000]	0.969 [0.948; 0.990]	0.976 [0.960; 0.992]	0.985 [0.971; 1.000]	0.983 [0.968; 0.998]	0.966 [0.940; 0.981]	0.972 [0.949; 0.984]
Observer 7	0.990 [0.980; 1.000]	0.994 [0.987; 1.000]	1.000 [1.000; 1.000]	0.987 [0.968; 1.000]	0.993 [0.981; 1.000]	1.000 [1.000; 1.000]	1.000 [1.000; 1.000]	1.000 [1.000; 1.000]	1.000 [1.000; 1.000]	1.000 [1.000; 1.000]	0.953 [0.917; 0.973]	0.904 [0.830; 0.945]
Observer 8	1.000 [1.000; 1.000]	1.000 [1.000; 1.000]	1.000 [1.000; 1.000]	1.000 [1.000; 1.000]	1.000 [1.000; 1.000]	1.000 [1.000; 1.000]	1.000 [1.000; 1.000]	1.000 [1.000; 1.000]	1.000 [1.000; 1.000]	1.000 [1.000; 1.000]	0.994 [0.990; 0.997]	0.990 [0.983; 0.995]
Observer 9	0.982 [0.963; 1.000]	0.996 [0.988; 1.000]	1.000 [1.000; 1.000]	0.989 [0.966; 1.000]	1.000 [1.000; 1.000]	0.958 [0.916; 1.000]	0.989 [0.978; 1.000]	0.984 [0.967; 1.000]	0.987 [0.973; 1.000]	1.000 [1.000; 1.000]	0.995 [0.991; 0.997]	0.992 [0.986; 0.996]
* Intra-observer reliability for the Kullman, Demirjian and GHK methods was assessed using weighted Cohen’s Kappa whereas Cameriere I3M method it was evaluated using the IntraClass Correlation Coefficient												

Overall inter-observer reliability was observed for I3M with ICC value of 0.986 [95% CI 0.980; 0.990]. When evaluating overall inter-observer reliability between staging methods, the DEM method achieved the highest Gwet’s AC2 value (0.918 [95% CI 0.910; 0.925]), followed closely by the GHK method (0.914 [95% CI 0.906; 0.923]), and the KUL method (0.868 [95% CI 0.849; 0.886]). A similar trend was observed in which the maxillary third molars exhibited lower agreement values in both DEM and GHK. Specifically, the maxillary and mandibular third molars in DEM method recorded Gwet’s AC2 values of 0.848 [95% CI 0.831; 0.864] and 0.936 [95% CI 0.926; 0.946], respectively. Likewise, in GHK method, the maxillary third molars demonstrated lower Gwet’s AC2 values (0.870 [95% CI 0.851; 0.888]) compared to the mandibular third molars (0.930 [95% CI 0.921; 0.938]). In the ICC calculation for I3M, a similar pattern was observed for both FDI 38 and FDI 48, with each tooth achieving the same ICC value (0.986 [95% CI 0.978; 0.991] and 0.986 [95% CI 0.978; 0.992], respectively). Detailed observations for each method are presented in Tables 4, 5, 6 and 7.

**Table 4:** Inter-observer reliability of the Kullman method

Inter-observer Reliability Kullman	Gwet’s AC2 [95% CI]	Total number of observations	*p*
Overall	0.868 [0.849; 0.886]	896	< 0.001
FDI 38	0.869 [0.849; 0.890]	449	< 0.001
FDI 48	0.867 [0.835; 0.899]	447	< 0.001

**Table 5:** Inter-observer reliability of the Demirjian method

Inter-observer Reliability Demirjian	Gwet’s AC2 [95% CI]	Total number of observations	*p*
Overall	0.918 [0.910; 0.925]	1744	< 0.001
Maxillary	0.848 [0.831; 0.864]	858	< 0.001
FDI 18	0.852 [0.827; 0.877]	429	< 0.001
FDI 28	0.843 [0.820; 0.867]	429	< 0.001
Mandibular	0.936 [0.926; 0.946]	896	< 0.001
FDI 38	0.935 [0.919; 0.952]	450	< 0.001
FDI 48	0.937 [0.923; 0.950]	446	< 0.001

**Table 6:** Inter-observer reliability of the Gleiser and Hunt method as modified by Köhler

Inter-observer Reliability Gleiser and Hunt as modified by Köhler	Gwet’s AC2 [95% CI]	Total number of observations	*p*
Overall	0.914 [0.906 0.923]	1753	<0.001
Maxillary	0.870 [0.851; 0.888]	858	<0.001
FDI 18	0.874 [0.848; 0.900]	428	<0.001
FDI 28	0.866 [0.838; 0.893]	430	<0.001
Mandibular	0.930 [0.921; 0.938]	895	<0.001
FDI 38	0.933 [0.923; 0.942]	449	<0.001
FDI 48	0.926 [0.913; 0.940]	446	<0.001

**Table 7:** Inter-observer reliability of the Cameriere I3M method

Inter-observer Reliability Cameriere I3M	ICC [95% CI]	Total number of observations	*p*
Overall	0.986 [0.980; 0.990]	864	<0.001
FDI 38	0.986 [0.978; 0.991]	441	<0.001
FDI 48	0.986 [0.978; 0.992]	423	<0.001

As shown in Table 8, the analysis of agreement on NA responses revealed the highest Gwet’s AC1 values for the KUL method (0.993 [0.982; 1.000]) and the I3M method (0.988 [0.974; 1.000]), followed by the DEM and GHK methods, which achieved values of 0.954 [95% CI 0.936; 0.973] and 0.954 [95% CI 0.935; 0.973], respectively. These high Gwet’s AC1 values indicate a high level of agreement among observers concerning teeth that cannot be assessed. Furthermore, the analysis highlighted that mandibular molars demonstrated higher reliability for NA responses, with values of 0.994 [95% CI 0.983; 1.000] in DEM and 0.992 [95% CI 0.980; 1.000] in GHK, compared to maxillary molars, which showed lower reliability (0.911 [95% CI 0.874; 0.948] in DEM and 0.913 [95% CI 0.876; 0.951] in GHK).

**Table 8:** Inter-observer reliability on Not applicable (“NA”) teeth responses

Inter-observer Reliability NATeeth	Gwet’s AC1 [95% CI]	Total number of observations	*p*
Kullman	0.993 [0.982; 1.000]	900	< 0.001
Demirjian (overall)	0.954 [0.936; 0.973]	1800	< 0.001
Maxillary	0.911 [0.874; 0.948]	900	< 0.001
Mandibular	0.994 [0.983; 1.000]	900	< 0.001
Gleiser and Hunt as modified by Köhler (overall)	0.954 [0.935; 0.973]	1800	< 0.001
Maxillary	0.913 [0.876; 0.951]	900	< 0.001
Mandibular	0.992 [0.980; 1.000]	900	< 0.001
Third Molar Maturity Index (I3M)	0.988 [0.974; 1.000]	900	< 0.001

All observers who were allowed to use image processing software to aid in the process reported using ImageJ (version 1.49), an open-source computer-aided drafting program designed for processing and analyzing digital X-ray images.

## Discussion

Assessing the reliability of a methodology is a critical step in research, particularly when the method involves a degree of subjectivity [11]. This aspect of reliability is essential to ensure that a measurement tool (in this case, the staging method) performs as intended and does so consistently each time it is used [31]. This is particularly relevant for third molars, an anatomical region known for its challenging anatomical positions and its role in age estimation for disputed age cases, where achieving high levels of agreement is especially crucial. In this study, three major staging methods were examined alongside an additional calibration guideline, and the results demonstrated that all nine observers achieved high consistency and reliability, with intra-observer reliability values exceeding 0.8, indicative of strong individual agreement.

Consistently lower agreement was observed for maxillary third molars compared to mandibular third molars across the various staging methods. The maxillary third molars received lower scores in both the DEM and GHK methods, with Gwet’s AC2 values for the maxillary third molars in DEM (0.848) and GHK (0.870) being significantly lower than those for the mandibular third molars (DEM = 0.936, GHK = 0.930). Previous literature [32, 33] has shown that maxillary third molars tend to present lower reliability, which aligns with the findings of this study, where the maxillary third molars consistently received lower agreement values for both the DEM and GHK methods. Similarly, Nguyen et al. [34] observed lower agreement for third molars in the maxillary jaw, suggesting that improvements in imaging quality or enhanced observer training could help mitigate these challenges and improve staging accuracy. de Oliveira Santos et al. [35] demonstrated a greater degree of discrepancy between the classification of maxillary and mandibular third molars. In contrast, Uys et al. [36] reported higher inter-observer agreement for maxillary third molars compared to mandibular ones.

This finding suggests that practitioners may face greater challenges in accurately assessing maxillary third molars, likely due to anatomical variability and their positioning, which can affect both visibility and accessibility during evaluation. For instance, the maxillary third molar region is often obscured by superimpositions such as ghost shadows from the contralateral mandible and overlapping structures of the maxillary sinus [37].

Other studies proposed to assess the third molar staging using a three-dimensional imaging, such as Cone Beam Computed Tomography (CBCT) or Magnetic Resonance Imaging (MRI) [38, 39]. However, it should be noted that staging methods developed for two-dimensional imaging (i.e., staging that were used in this study) needs to be recalibrated for three-dimensional modalities, as changes in modality may pose challenges for observers. For example, Franco et al. [40] reported significantly lower third molar staging agreement in CBCT compared to panoramic radiographs [40]. This highlights the potential need for enhanced clinical staging guidelines or additional guidance specifically for staging maxillary third molars. 

The overall inter-observer reliability gives the highest Gwet’s AC2 value for DEM (0.918), closely followed by the GHK (0.914), and KUL showing the lowest value (0.868). These results indicate that the DEM and GHK staging methods yield greater inter-observer reliability compared to the GHK and KUL methods. However, this does not imply that the DEM method should be applied every time where third molar staging is required. The marginal differences observed between the DEM and other methods suggest that both strategies can be used effectively in clinical practice together; nevertheless, the slightly better agreement associated with DEM staging may favor its broader adoption.

This discrepancy across stages may be attributable to variations in observer training, the population studied, or the study methodology. Observers might have been more familiar with the DEM or GHK staging methodology. However, the reliability of KUL observed in this study remained at an acceptable level, and potential improvements in observer instruction or the standardization of evaluation parameters could help reduce this gap in comparison to the DEM and GHK methods [41, 42].

Even with similar performance, choosing KUL over DEM or GHK can lead to a data restriction due to KUL staging does not have crown development staging. Therefore, choosing a staging needs to be tailored to the purpose of the study [43]. For example, studies with the purpose of estimating an individual legal age (i.e., above or below 18 years old) needs to utilise a staging method with higher apical closing division (i.e., GHK).

Among the methods evaluated in this study, the I3M stands apart due to its reliance on a continuous metric measurement rather than on ordinal stages. Although its ICC values indicated excellent agreement, achieving perfect reliability remains challenging. Even with careful application of the method, repeated measurements of the same tooth may yield slightly different I3M values (i.e., 0.812 vs. 0.804), as minor variations in cursor placement during third molar measurement can produce small differences in the final result. In contrast, staging based systems such as DEM, GHK, or KUL, once a tooth stage is noted — for instance, stage D — subsequent assessment by the same or another observer almost invariably assign the identical stage, provided the criteria are applied correctly and the observer is calibrated. Similar observations were reported by Thevissen and colleagues [43, 44]. Thevissen et al. [44] stated that third molar scorings (categorical data) are best related to age and provide the most accurate age predictions compared to all collected tooth measurements and ratios of tooth measurements (continuous data). The authors noted that tooth measurements may exhibit greater variability compared to ordinal staging systems, even when ratio corrections are calculated to take into account the variation in the radiograph and tooth size in every individual [43]. Moreover, even when the two methods are combined, no significant increase in the accuracy of age estimation is observed. Therefore, when both approaches are feasible, an ordinal staging system may be preferred in forensic practice, as it provides high reliability and is less sensitive to subtle measurement differences.

In the current study, NA values were calculated to quantify inter-observer agreement regarding the decision to use an adjacent tooth, particularly in instances where the primary tooth under observation could not be staged. The KUL method exhibited the highest Gwet’s AC1 values (0.993), followed by I3M (0.988), and both DEM and methods (0.954 each) (Table 6). This finding is consistent with observations from other studies [45–48], who highlighted the KUL and I3M methods, as particularly effective in handling ambiguous cases in which tooth staging was indeterminate. Additionally, Garamendi et al. [49] reported that the KUL method demonstrated higher agreement in identifying cases where teeth could not be accurately staged due to anatomical variations, which may explain its superior performance in the present study with respect to “NA” responses.

Other studies [50, 51] have also demonstrated that the reliability of NA responses for mandibular molars is significantly greater than for maxillary molars, consistent with the findings of the present study. In this study, mandibular third molars showed higher reliability values in DEM and GHK (0.994 and 0.992, respectively) compared to maxillary third molars (0.911, 0.913). This difference is likely due to the more accessible position of mandibular molars in the jaw, making them easier to assess with greater precision.

A limitation of this study was the inclusion of only four specific DAE methods. Other established staging methodologies with a greater number of developmental stages (e.g., Moorrees, Nolla) should also be investigated, as the number of stages may influence both inter- and intra-observer reliability [52, 53]. Additionally, although the statistical measures used in this study (i.e., Cohen’s Kappa, Gwet’s AC2, ICC) provide quantitative estimates of reliability, they do not directly translate into real-life scenarios of scientific decision-making. Despite demonstrating high statistical agreement, a given method may still be prone to misclassification at the level of individual ages, influenced by factors including observer expertise and imaging modality.. The inclusion of the Cameriere I3M metric method [26] alongside three stage-based methods was intended to provide a broader overview of commonly applied approaches in DAE. Although recent studies have undertaken comparisons between these methodologies, the fundamental methodological differences between metric and stage-based assessments necessitate that direct comparisons be interpreted with caution.

Although the study sample comprised 50 individuals, each of the four third molars was assessed separately with both the DEM and GHK staging methods, thereby increasing the overall number of assessments. Nevertheless, the relatively small number of individuals limits both the generalizability and the statistical precision of the findings, particularly with respect to infrequently observed stages. The findings of this study should be generalized with caution, taking into account the specific characteristics of the sample. Future studies employing larger, more diverse and representative samples are warranted to confirm the applicability of these findings across other contexts. In fact, previous studies on this topic [54], indicate that the greatest gains in precision (i.e., largest change in Mean Standard Error values) can be achieved by increasing a small initial sample size or a small number of repeated measures. For example, increasing the number of observers from 2 to 3 yields a greater gain in precision than increasing from 4 to 5, just as increasing the sample size from 10 to 20 produces more improvement than increasing from 40 to 50. In addition, as measurements can be expensive and burdensome to practitioners and researchers, we do not recommend collecting more data than required to assess intra- and inter-rater reliability values as this would lead to research waste [55]. This therefore suggests basing the sample size calculations should be based on achieving sufficient precision of the estimate, specifically with regard to the width of the 95% CI.

It should be emphasized that Fleiss’ kappa was not reported alongside Gwet’s AC1. Although the proportion of agreement in each instance exceeded 90%, the kappa statistic yielded artificially low coefficients. Accordingly, Gwet’s AC1 was adopted, given its reduced sensibility to prevalence effects. Nevertheless, as highlighted by Vach and Gerke [56], AC1 values warrant circumspect interpretation, particularly given that Landis and Koch [29] benchmarking approach was designed for kappa-based methods.

The results of this investigation align with previous studies [43, 57], confirming that DEM and GHK remain two of the most reliable methods for third molar staging. Furthermore, the consistently lower agreement for maxillary third molars and higher agreement for mandibular molars compared to maxillary first molars is expected, given the anatomical challenges associated with maxillary molar teeth, as noted in the existing literature. The higher Gwet’s AC1 for “NA” responses in both the KUL and I3M methods further supports the robustness of these techniques in handling ambiguous cases.

In summary, forthcoming studies are expected to delve deeper into methodologies for staging maxillary third molars, while also exploring the potential contributions of advanced imaging techniques and artificial intelligence in enhancing observer agreement. Despite these limitations, it remains crucial to investigate inter-observer variability across larger cohorts when applying DAE methods for forensic purposes, as well as to validate the methods in populations beyond those used for their initial calibration. It is imperative that forensic personnel have confidence in the robustness, reliability, and accuracy of the method for it to be deemed admissible in legal proceedings, while also recognizing the level of expertise and training required for its appropriate application.

The findings of this study demonstrate that experts in the field can achieve highly comparable assessments, and that the application of any technique requires validation through studies of this kind to ensure greater reliability in decision-making.

## Conclusions

The methods tested for reliability in third molar staging and measurement yielded overall highly reproducible results, although some differences were observed between maxillary and mandibular third molars, with the maxillary third molars being more prone to disagreement. Within the staging methods, the DEM demonstrated the highest overall reliability. However, it should be noted that the wide intervals between stages may compromise the accuracy of assessments, particularly for the apical closure stages. Additionally, the staging method selected for a study should align with the study's objectives, with methods incorporating multiple stages toward final stages of root and apical formation being particularly relevant for legal age assessment when the threshold of interest is 18 years old. Moreover, although the I3M method exhibited the highest reliability values, obtaining identical measurements across two separate observations was nearly impossible due to its metric approach.

## Data Availability

The data supporting this study’s findings are available on request from the corresponding author.
