# Correction to: Assessing observer-dependent dental age estimation procedures: intra- and inter-observer reliability across four well established radiographic systems for dental analysis

**DOI:** 10.1007/s00414-025-03671-3

**Published:** 2025-11-20

**Authors:** Nikolaos Angelakopoulos, Rizky Merdietio Boedi, Ademir Franco, Nikita Polukhin, Akiko Kumagai, Ivan Galic, Jeta Kelmendi, Israel Soriano Vázquez, Sang-Seob Lee, Galina Zolotenkova, Roberto Scendoni, Stefano De Luca

**Affiliations:** 1 Department of Orthodontics and Dentofacial Orthopedics, University of Bern, Bern, Switzerland; 2 Department of Forensic Dentistry, Faculdade São Leopoldo Mandic, Campinas, Brazil; 3 Department of Dentistry, Faculty of Medicine, Universitas Diponegoro, Semarang, Indonesia; 4 Department of Public Health and Medical Social Sciences, Synergy University, Moscow, Russian Federation; 5 Department of Forensic Science, Division of Forensic Odontology and Disaster Oral Medicine, Iwate Medical University, Iwate, Japan; 6 Department of Oral Surgery, School of Medicine, University Hospital of Split, University of Split, Split, Croatia; 7 Department of Orthodontics, University Dentistry Clinical Center of Kosovo, Pristina, Kosovo; 8 Forensic Odontologist, independent researcher, Mexico City, Mexico; 9 Department of Anatomy, Catholic Institute for Applied Anatomy, College of Medicine, The Catholic University of Korea, Seoul, Seocho-gu Republic of Korea; 10 Department of Forensic Medicine, I.M. Sechenov First Moscow State Medical University, Moscow, Russian Federation; 11 Department of Law, Institute of Legal Medicine (AgEstimation Project), University of Macerata, Macerata, Italy; 12 Department of Organisms and Systems Biology, Faculty of Biology, University of Oviedo, Oviedo, Spain

**Correction to: International Journal of Legal Medicine**

10.1007/s00414-025-03616-w

In the original version of the manuscript, there was a discrepancy between the article title in the web version and in the online PDF due to the outdated XML that was uploaded.

The correct article title should be “Assessing observer-dependent dental age estimation procedures: intra- and inter-observer reliability across four well established radiographic systems for dental analysis”.

Relevant corrections in the body text were also updated in the XML to ensure the web version is identical to the Online PDF.

The original article has been corrected.

